# Ballooned neurons in semi-recent severe traumatic brain injury

**DOI:** 10.1186/s40478-023-01516-x

**Published:** 2023-03-10

**Authors:** Jean Michaud, Isabelle Plu, Jacqueline Parai, André Bourgault, Caroline Tanguay, Danielle Seilhean, John Woulfe

**Affiliations:** 1 Department of Pathology and Laboratory Medicine, University of Ottawa, Ottawa, Canada; 2 Raymond Escourolle Département de Neuropathologie, Hôpital Pitié-Salpêtrière, APHP, Université de La Sorbonne, Paris, France; 3 Institut Médico-Légal, Paris, France; 4 Eastern Ontario Forensic Pathology Unit, University of Ottawa, Ottawa, Canada; 5 Laboratoire de Sciences Judiciaires Et de Médecine Légale, Montréal, Québec Canada; 6 Department of Pathology and Laboratory Medicine, The Ottawa Hospital, University of Ottawa, Ottawa, Canada; 7 Program in Neuroscience, Ottawa Hospital Research Institute, Ottawa, Canada

**Keywords:** Ballooned neurons, Traumatic brain injury, Traumatic axonal injury, Proximal axonal swellings, Neurodegeneration

## Abstract

Traumatic brain injury (TBI) is now recognized as an insult triggering a dynamic process of degeneration and regeneration potentially evolving for years with chronic traumatic encephalopathy (CTE) as one major complication. Neurons are at the center of the clinical manifestations, both in the acute and chronic phases. Yet, in the acute phase, conventional neuropathology detects abnormalities predominantly in the axons, if one excludes contusions and hypoxic ischemic changes. We report the finding of ballooned neurons, predominantly in the anterior cingulum, in three patients who sustained severe TBI and remained comatose until death, 2 ½ weeks to 2 ½ months after the traumatic impact. All three cases showed severe changes of traumatic diffuse axonal injury in line with acceleration/deceleration forces. The immunohistochemical profile of the ballooned neurons was like that described in neurodegenerative disorders like tauopathies which were used as controls. The presence of αB-crystallin positive ballooned neurons in the brain of patients who sustained severe craniocerebral trauma and remained comatose thereafter has never been reported. We postulate that the co-occurrence of diffuse axonal injury in the cerebral white matter and ballooned neurons in the cortex is mechanistically reminiscent of the phenomenon of chromatolysis. Experimental trauma models with neuronal chromatolytic features emphasized the presence of proximal axonal defects. In our three cases, proximal swellings were documented in the cortex and subcortical white matter. This limited retrospective report should trigger further studies in order to better establish, in recent/semi-recent TBI, the frequency of this neuronal finding and its relationship with the proximal axonal defects.

**Supplementary Information:**

The online version contains supplementary material available at 10.1186/s40478-023-01516-x.

## Introduction

Traumatic brain injury (TBI) is a major cause of disability and death affecting all ages, worldwide. From the mild concussion symptoms of the acute phase to the cognitive decline and dementia years after injuries, the range of cerebral morphological lesions and affected cellular pathways is diverse and our understanding of these remains incomplete [1]. It is now recognized that TBI is not a static insult with repair and associated sequelae but a dynamic process of degeneration and regeneration potentially evolving for years, particularly in moderate to severe TBI [1–4]. Neurons are at the center of the clinical manifestations both in the acute phase (loss of consciousness, dizziness, visual disturbances, and coma) and chronic phase, the latter with several comorbidities including various neurodegenerative disorders [5] and chronic traumatic encephalopathy (CTE) [6]. Yet, in the acute phase, conventional neuropathology detects changes attributable to trauma in axons, but the neuronal cell body remains relatively intact if one excludes contusions and hypoxic-ischemic encephalopathy [3, 7, 8]. This report is driven by the incidental finding of ballooned neurons, predominant in the cingula, in three cases of semi-recent severe TBI.

## Clinical history

Case 1. This 32-year-old man was involved in a bicycle-car accident. In the emergency room, his Glasgow Coma Scale (GCS) was 3–4 with signs of elevated intracranial pressure (ICP). He underwent a full trauma CT-scan which disclosed bilateral subdural hematomas, cerebral edema, comminuted skull fractures of the temporal and frontal bones and fractures of the first rib and clavicles. No injuries were reported to his abdomen or pelvis. No trace of alcohol was found in his blood. He was diagnosed with severe head injuries and transferred to the intensive care unit. The initial management was conservative with insertion of an intracranial pressure (ICP) monitor and management for his raised ICP. His ICP, despite all medical treatment, remained high. A repeat CT-scan of the head showed worsening cerebral edema and an increase in the size of the left subdural hematoma. He was taken to the operating room where he underwent bilateral frontal craniectomies, evacuation of the left subdural hematoma, right frontal partial lobectomy (contusion/laceration), and insertion of an external ventricular drain. Despite medical and surgical interventions, he experienced persistently elevated intracranial pressure, low GCS, and no evidence of neurological improvement. He died 16 days after admission.

At postmortem examination there was evidence of prior bifrontal craniectomies with persistent herniation of the frontal lobes. Several skull fractures were noted at the base of the skull. Anteriorly there were several fractures involving the left orbital roof of the frontal bone. A linear fracture also extended diagonally across the anterior cranial fossa, involving the ethmoid bone and the right orbital roof of the frontal bone. Within the middle cranial fossa there were linear fractures involving the right side of the sphenoid and temporal bones, and of the left temporal bone. The fractures of the left first rib and both clavicles were confirmed. Acute bronchitis was present in the upper lobe of the right lung. There was also evidence of a contusion in the right middle lobe with extensive intra-alveolar hemorrhage. The cause of death was attributed to head injuries.

Case 2. This 83-year-old man, without past-medical history except for suspicion of alcoholism, was found unconscious near a bar. In the emergency department, he was comatose (GSC 3). Recent wounds or ecchymoses of the left and right frontal, left temporal, and left mastoid regions were documented. Bilateral peri-orbital edema was present. He also had a laceration of the left lower lip and an abrasion of the vertex. His blood alcohol level was 35.6 mmol/L (164 mg/100 ml). The initial CT-scan of the head and neck showed fractures of the nasal bone, left maxillary sinus, and left zygomatic bones. There was no evidence of acute ischemic/hemorrhagic lesions or contusions. Bilateral hygromas and leukoaraiosis of the white matter were noted. Osteo-degenerative changes were present at the cervical level without evidence of fracture. As the nature of his persistent deep coma was nebulous, an MRI was performed. Beyond the findings already noted with the CT-scan, there were minute lacunes in the genu of the left internal capsule, right external capsule, and pons. Carotid angiograms excluded a dissection. A second MRI was performed 19 days after admission. No significant changes were noted except for a small enhancing focus, (possibly a hemosiderin deposit), in the right superior frontal gyrus. A gastrostomy and tracheostomy were performed. He remained in a vegetative state until his death due to bronchopneumonia 2 ½ months after his admission.

The post-mortem findings included: pulmonary edema with focal bronchopneumonia, severe coronary atherosclerosis, remote myocardial infarct, healed fractures, and cutaneous lesions, the topography of which are described above. There was no evidence of skull fracture or intracranial hemorrhage. The overall clinical and morphological findings were consistent with a blunt cranio-cerebral injury as the favoured mechanism.

Case 3. This 33-year-old man was the victim of an assault. His past medical history included hepatitis C and HIV infections. On admission, a GCS of 5 was documented with several craniofacial lacerations/contusions in the left orbital region, right frontal region (with hematoma) and occipital region. A CT-scan of the head showed cerebral edema, right frontal and left posterior temporal contusions, a left 7 mm-thick subdural hematoma with shift of the midline to the right, early herniation of the temporal unci and diffuse subarachnoid hemorrhage. A decompressive left craniectomy with evacuation of the subdural hematoma was performed. His overall status did not improve, his GCS remaining at 3. After discussion with his family, he was extubated and died five days later, three weeks after the traumatic incident.

The post-mortem findings included healing external contusion/lacerations, aspiration pneumonia and hepatic cirrhosis. Findings at the level of the craniectomy site are detailed below in the neuropathology description. The cause of death was attributed to blunt cranio-cerebral injuries.

## Material and methods

Authorizations for reporting these three cases were granted by the Eastern Ontario Regional Forensic Unit and the Laboratoire de Sciences Judiciaires et de Médecine Légale du Québec.

The sampling followed a relatively standardized protocol for all TBI cases: samples were collected from the cortex and underlying white matter of the pre-frontal gyrus, superior and middle frontal gyri, temporal pole, parietal and occipital lobes, deep frontal white matter, hippocampus, anterior and posterior corpus callosum with the cingula, lenticular nucleus, thalamus with the posterior limb of the internal capsule, midbrain, pons, medulla, cerebellar cortex and dentate nucleus. In some cases, gross pathology (e.g. contusions) mandated further sampling along with the dura and spinal cord if available. The number of available sections for these three cases was 26 for case1, and 24 for cases 2 and 3.

For the detection of ballooned neurons, all HE or HPS sections, including contusions, were screened at 200×.

Representative sections were stained with either hematoxylin–eosin (HE) or hematoxylin-phloxin-saffron (HPS). The following histochemical stains were used: iron, Luxol-periodic acid Schiff (Luxol-PAS) and Bielschowsky. The following antibodies were used for immunohistochemistry: glial fibrillary acidic protein (GFAP) (Leica, PA0026,ready to use), CD-68 (Leica, PA0073, ready to use), neurofilament 200 (NF200) (Leica, PA371, ready to use), beta-amyloid precursor-protein (β-APP) (Chemicon/Millipore, MAB348, 1/5000), αB-crystallin (EMD Millipore, MABN2552 1/1000), ubiquitin (Vector, 1/400), β-amyloid (Dako/Agilent, 1/100), tau protein (Thermo/Fisher, MN1020 1/2500), synaptophysin (Dako/Agilent, ready to use), TAR DNA binding protein 43 (TDP-43) ((Protein Tech, 10,782-2AP, 1/50), fused in sarcoma binding protein (FUS) (Protein tech, 60,160–1-1 g, 1/100), and p62 (BD Transduc, 1/25). In our index cases, the following were used for the evaluation of TAI: β-APP, GFAP, CD68 and NF200; for the neurodegenerative changes: αB-crystallin, NF200, ubiquitin, tau protein, synaptophysin, TDP-43, FUS were used.

For the characterization of the ballooned neurons only, two cases of fronto-temporal lobar degeneration, FTLD-Tau, were used as controls. One was a female aged 72 who presented with speech difficulties followed by neurocognitive decline and eye movement abnormalities raising the possibility of Richardson’s disorder. The other was a male aged 67 who presented with a primary non-fluent aphasia progressing to fronto-temporal demαentia. In both cases, the morphological findings were characteristic of a corticobasal degeneration.

## Neuropathological findings

### Gross description

Case 1. The brain weighed 1860 g after fixation. Subdural hematomas were present bilaterally, most extensive on the left side with a thickness of 0.1–0.2 cm. Variable brown discoloration was indicative of ongoing resorption. Bilaterally, severe herniation of cerebral tissue was noted at the level of the craniectomies. The left hemisphere was more voluminous than the right. Changes consistent with a partial frontal lobectomy were found on the right side. Contusions/lacerations were noted in the right frontal and temporal lobes and on the orbital surface of the left frontal lobe. A subpial 0.5 cm hemorrhage was present in the left middle frontal gyrus. In all of these lesions, xanthochromia was present, indicative of resorption. Bilateral temporal uncal herniation and prominent cerebellar tonsils were noted. The cut sections confirmed the above. Additional findings included bilateral ventricular drain tracts, irregular gray softening of the posterior temporal lobe white matter, splenium of the corpus callosum and under the falx cerebri in the genu of the corpus callosum, rupture of the septum pellucidum, prominence of the white matter vasculature and punctate hemorrhages in the tegmentum of the pons.

Case 2. The brain weighed 1300 g prior to fixation. External examination revealed diffuse cortical atrophy. There was no evidence of contusion but a small xanthochromic focus was noted in the arachnoid of the infero-lateral left occipital lobe. The subdural compartment showed small foci of blood in the left frontoparietal and left orbitofrontal regions. There was no atherosclerosis of the arteries at the base of the brain. The cut sections revealed atrophy of the cortical gyri to be moderate with attendant moderate enlargement of the ventricular system. The anterior commissure appeared atrophic as well. A lacunar infarct was noted in the subcortical region of the left frontal lobe.

Case 3. The brain weighed 1480 g prior to fixation. Changes associated with the left craniectomy were present with evidence of recent rebleeding in the subdural space and presence of a moderate amount of blood in the epidural space. Residual blood was partly xanthochromic in color. A thin layer of subdural hemorrhage, again with xanthochromia, was also noted along the falx cerebri, bilaterally, over the parieto-occipital convexity on the right side and over the tentorium cerebelli on the left side. The cerebral hemispheres were asymmetrical with signs of external compression involving the frontal, parietal and temporal lobes with shift of the midline structures to the right. A left temporal contusion involved its pole and inferior surface. Subpial petechiae were present in both the orbitofrontal and right temporal lobe region. The cut sections confirmed the signs of external compression of the left hemisphere with shift of the midline structures to the right and early left uncal herniation. A small 0.8 cm hematoma was noted in the superficial left posterior temporal lobe. Petechiae were present in the lateral part of the genu of the corpus callosum and in the white matter of both frontal lobes.

### Histology

**Fig. 1:**
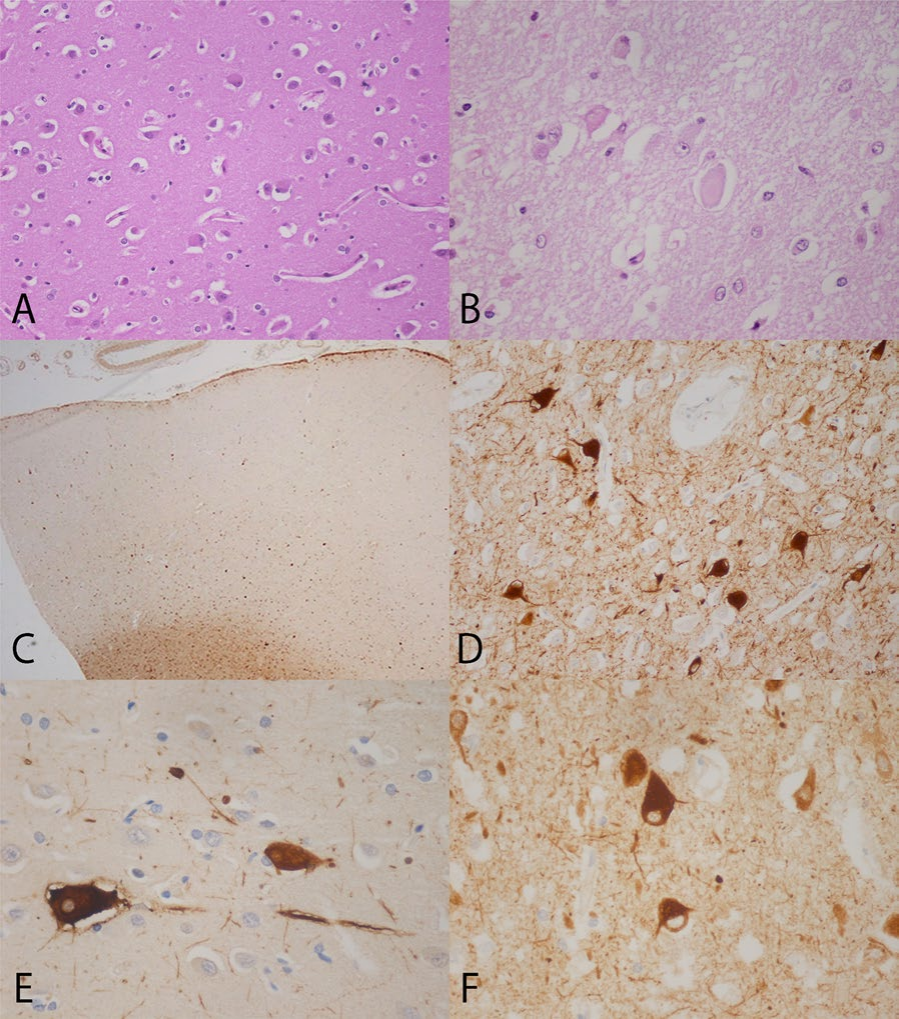
Histology and immunohistochemistry of ballooned neurons in cortex of the cingulum. **a** View of cortex in the cingulum showing several ballooned neurons, in Case 3 (HPS, ×200). **b** High power view showing one ballooned neuron with inclusion-like appearance, in Case 1 (HPS). **c** Low power view of the cingulum in Case 3 showing greater number of ballooned neurons in layers 4–6 of the cerebral cortex, without topographical pattern (αB-crystallin, ×50). **d** High power view of αB-crystallin showing strong positivity in ballooned neurons and their projections as well as positive neuropil thread, in Case 1 (αB-crystallin, ×400). **e** In the cortex of the cingulum, in Case 1, αB-crystallin positive ballooned neurons, one with cleft and positive rim of astrocytic processes; positive neuropil threads are also seen (αB-crystallin, ×600). **f** Positivity of ballooned neurons with NF200, in Case 1 (NF200, ×600)

**Fig. 2:**
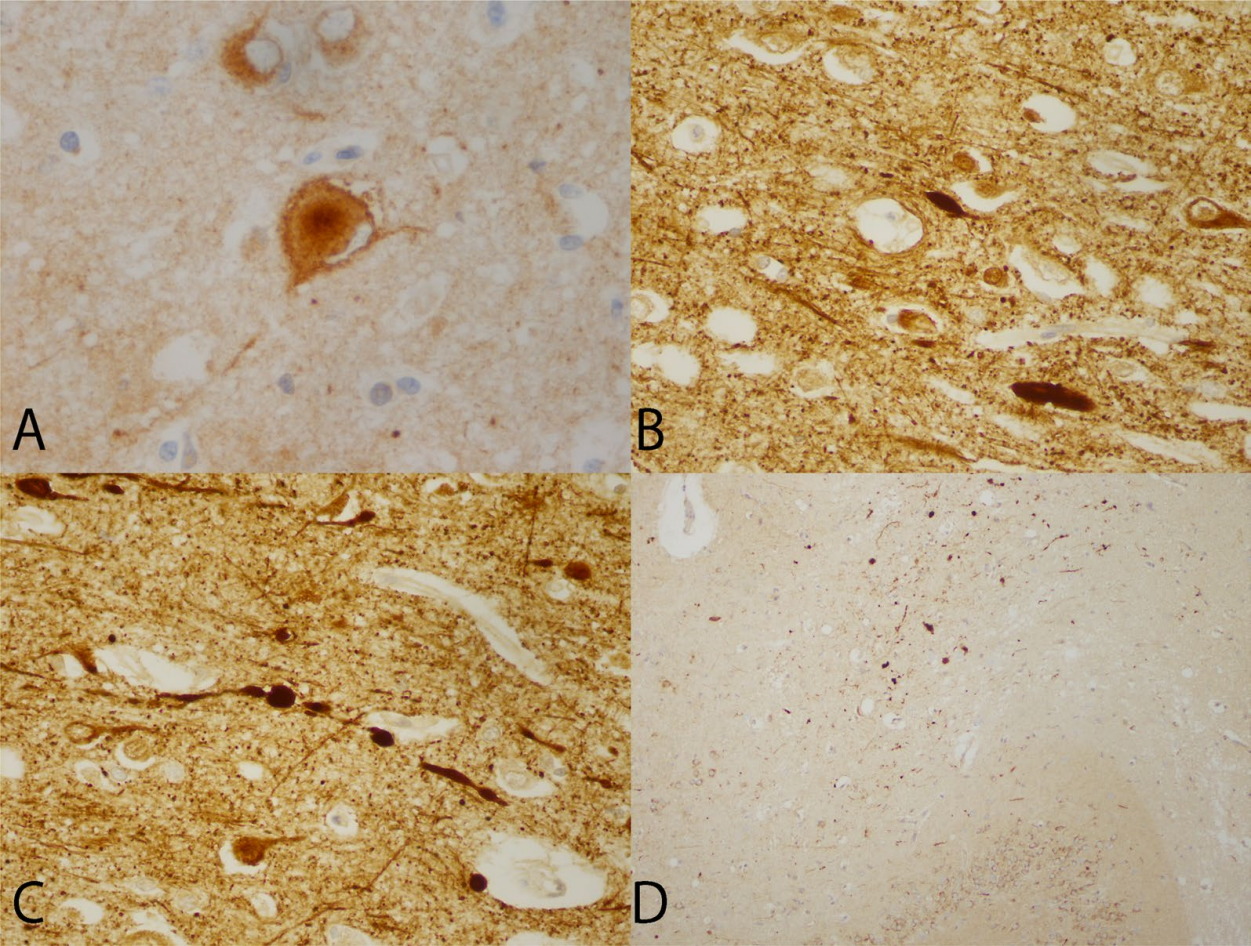
Proximal axonal swellings and inclusion-like structure in ballooned neuron. **a** β-APP positive ballooned neuron in the cingulum of Case 1, with inclusion-like appearance (β-APP, ×600). **b** NF-200 immunohistochemistry of cingulum in Case 1 showing axonal swellings (NF-200, X400). **c** NF200 of cortex of cingulum showing a beaded axon (NF200, ×400). **d** Low power view of striatum radiatum and lacunosum of Case 1 with numerous small axonal swellings (β-APP, ×100)

Common findings in all cases: In the cerebral cortex of the cingula, there were several ballooned neurons characterized by marked enlargement and eccentric nuclei (Fig. 1a). In some cells, a peripheral layer of vestigial Nissl substance was present, giving the impression of a cytoplasmic inclusion (Fig. 1b). Some neurons displaying mild swelling suggestive of a transition from normal to the ballooned status were also noted. The ballooned neurons were found predominantly in the fourth to six layers of the cortex (Fig. 1c) although they could be seen up to the second layer. Although their distribution showed this laminar predilection, they did not display any topographic predilection with respect to sulcal crests versus depths, perivascular regions, etc. They were significantly less numerous in the mid and posterior cingula. Immunochemistry showed αB-crystallin positivity in the perikarya of ballooned neurons, with frequent extension into the hillock and dendrites, as well as in numerous neuropil threads (Fig. 1d). AlphaB-crystallin staining was present in some oligodendrocytes and astrocytes, as described previously [10]. In some instances, clefts between the perikarya and the neuropil were present and the αB-crystallin immunostaining showed positivity of the rim around these clefts, likely representing astrocytic processes (Fig. 1e). The following antibodies also produced positive staining in the cytoplasm of ballooned neurons: NF200, β-APP, and ubiquitin. NF200 (Fig. 1f) positivity was also noted in the cytoplasm of most cortical neurons. β-APP staining produced, in some neurons, the impression of an inclusion body (Fig. 2a), and detected small axonal swellings in the deeper cortex (Fig. 2b) as well as in the underlying subcortical white matter. The β-APP and the NF200 stains also showed very rare axons with beading (Fig. 2c). Staining using the following antibodies was not contributory: synaptophysin, tau protein, β-amyloid, TDP-43 and FUS.

The number of ballooned neurons in the cingula was greatest in Case 1, intermediate in Case 3 and least in Case 2. Ballooned neurons were also seen, always more often in Case 1, above the cingula, in the mesial fronto-parietal lobes. Less frequently, they were seen in the frontal convexities. In case 1, they were quite numerous in the temporal cortex. In Case 3, a few were noted in the temporal and parietal convexities. In the hippocampus, in Case 1, they were quite numerous at the junction between the CA4 and CA3 sectors. In the underlying striatum radiatum and lacunosum, small axonal swellings were detected with β-APP (Fig. 2d). This was not seen in the other two cases. In Case 1, rare swellings were noted in the subiculum and entorhinal cortex. In case 2, one was seen in the CA4 sector. In cases 1 and 2, rare examples were present in the thalamus and, in case 3, in the striatum. None were seen in the brainstem or cerebellum. The ballooned neurons were not associated with the contusions. The immunohistochemistry of ballooned neurons, done on our two FTLD- Tau cases used as controls, showed results similar to those described in our three patients.

All three cases showed changes of traumatic diffuse axonal injury. This was based on the presence of numerous β-APP positive axonal swellings in the corpus callosum, white matter of the frontal lobes, posterior limbs of the internal capsules, and brainstem. In case 2, consistent with the longer survival, there were also several clusters of microglial cells, mostly in the brainstem. Notwithstanding the fact that one patient survived 2 ½ months while the others survived 2 ½ weeks, there appeared to be, qualitatively, a correlation between the number of ballooned neurons and the number of cortical / subcortical proximal swellings. Also, the number of the latter correlated in general with the severity of the TAI in the white matter. Occasional resorbing petechiae were noted in the corpus callosum and dorsal quadrants of the upper brainstem in cases 1 and 3. Axonal swellings attributed to hypoxic-ischemic foci and lesions secondary to compression of the anterior cerebral arteries or upper midbrain by herniation were noted in case 1.

Histologic findings specific to each case (see Additional file 1).

## Discussion

Ballooned neurons, also sometimes referred to as enlarged, chromatolytic or achromatic, are found in a variety of disorders. It is known that disruption of axonal transport leads to accumulation of axonal cytoskeletal proteins between the nucleus and the axon hillock with displacement of Nissl bodies to the periphery of the neuronal cell body [9]. The accumulated proteins include ubiquitin-positive phosphorylated neurofilament epitopes [10] and the small heat shock protein αB-crystallin [10–13]. This protein is known to be upregulated in response to various pathological conditions associated with increased cellular stress, most notably those characterized by deficits in axonal transport, trans-neuronal degeneration and endoplasmic reticulum dysfunction. [10, 11, 14]. In this report, we postulate that the co-occurrence of diffuse axonal injury in the cerebral white matter and ballooned neurons in the cortex is mechanistically reminiscent of the phenomenon of chromatolysis. This has been most extensively studied in the spinal cord, experimentally and in humans, including in lower motor neuron disease. Ballooned neurons have been described in a subset of dementing neurodegenerative disorders [12, 13, 15]. They are particularly frequent in frontotemporal lobar degeneration (FTLD-tau) disorders including Pick’s disease and corticobasal degeneration which, interestingly, show prominent white matter/axonal involvement, when compared to another tauopathy, progressive supranuclear palsy, in which the white matter is relatively spared and the ballooned neurons not particularly numerous [7]. They are also seen in some cases of Alzheimer’s disease, argyrophilic grain disease, motor neuron disease, multiple system atrophy, Creutzfeldt-Jakob disease [10, 11, 15] and Lewy-body pathologies [16]. They are also present in pellagra, but, in this instance, ubiquitin staining was found to be negative [11]. One also notes the occasional finding of ballooned neurons in the vicinity of cortical infarcts or other focal lesions [11]. In human trauma, Cervos-Navarro and Lafuente reported central chromatolytic changes (without further characterization) rarely found in the somata of neurons in proximity of axonal damage at 48 h after the incident and lasting through the second post-traumatic week. Yet they commented on the notable absence, in most instances, of somatic or dendritic changes in the proximity of reactive/damaged axons [17].

Experimentally, in a model of traumatic axonal injury (TAI) generated by moderate central fluid percussion injury, neuronal somata linked to proximal axonal swellings were found to show an increase in β-APP positivity within two hours after the injury. These changes were detected in the neocortex, dentate gyrus, and thalamus. Interestingly, seven days later, the somata of some neurons displayed eccentric nuclei, interpreted by the authors as changes consistent with a chromatolytic response. These somatic changes were documented at the ultrastructural level, in the form of rough endoplasmic reticulum (RER) dispersion, nuclear eccentricity, cytoplasmic regions devoid of organelles, and clustering of the remaining ones. The cytoplasmic changes were noted as early as six hours after the injury with focal dispersion of the RER. At 24 h, they were well established and associated with Golgi dispersion. These changes were observed in neurons of variable size [18]. In assessing the proximity of the TAI in ballooned neurons, their data suggest that the lesion appears to occur in the proximal non-myelinated segment of the axon, immediately proximal to the first myelin segment, this site being considered a point of biomechanical vulnerability [18]. These findings were comparable to those observed in primary axotomy models, one notable difference being that this experimental model did not seem to lead to significant neuronal death. Furthermore, in the same fields, they noticed the presence of scattered necrotic neurons that were not axotomized, suggesting that neuronal somata could be damaged independently of axotomy [18].

In humans, this spatial relationship between proximal axonal disruption/swellings and chromatolytic changes in neuronal somata is found in motor-neuron disease. Axonal pathology with defective axonal transport is a key element in the multifactorial mechanisms culminating in motor neuron degeneration [19]. In amyotrophic lateral sclerosis, abnormal accumulation of phosphorylated neurofilaments, microtubules, mitochondria and lysosomes is detected in proximal axons and their swellings. The swollen perikarya also show abnormal accumulation of phosphorylated neurofilaments and various organelles [19]. Whether chromatolytic neurons can develop in the absence of proximal axonal dysfunction has not been established.

The presence of αB-crystallin positive ballooned neurons in the brain of patients who sustained severe craniocerebral trauma and remained comatose thereafter has never been reported. In the present study, they were found weeks/months after the traumatic events and were not in the vicinity of focal lesions. They were present predominantly in the cingula, anteriorly more than posteriorly. In addition, they displayed no specific topographical predilection like that described for tau inclusions in chronic traumatic encephalopathy [6] and no detectable abnormal deposition of tau protein. These findings, as reported herein, should be considered rare. A review of cases with similar clinical scenarios, in our two medical-legal neuropathology consultation services did not disclose additional cases. As the anterior cingulum is more and more recognized as a critical hub in cognitive and emotional functioning [20] and ideomotor mechanisms [21], the possibility that ballooned neurons in this region play a part in TBI symptomatology and its eventual complications, including CTE, is worthy of further study. In a recent meta-analysis, the anterior cingulum was recognized as one region of the brain involved in impaired self-awareness after TBI [22].

The overall clinical and morphological data of our three patients are consistent with angular acceleration-deceleration forces as the mechanism underlying cerebral damage. In this setting, the biological responses to the dynamic loads imposed on the head are, first, structural, represented by diffuse axonal injury (DAI) as the key pathogenic element and, second, functional with disrupted blood pressure [23]. DAI is predominantly localized to the midline; the most severely involved sites being the corpus callosum, the parasagittal white matter of the hemispheres (including the cingulum bundle), the internal capsules and the dorsolateral quadrants of the rostral brainstem. This is frequently associated with deep basal ganglia hematomas [23]. Consistent with this, in our cases, the neuronal changes predominated in the anterior cingula, a median site.

TAI/DAI, one of the most common neuropathological consequences of TBI, points to a selective vulnerability of white matter axons, damage resulting form the mechanical loading of the brain during acceleration/deceleration [24]. Experimental models and analysis of human post-mortem tissues provided evidence for a model consisting of the following sequence of events: axonal cytoskeletal disruption, disruption/interruption of axonal transport, swelling of the axon, disconnection, and wallerian degeneration [24]. The proposed mechanistic sequence leading to the development of the swellings includes primary breaking of axonal microtubules, undulation of the axons, and interruption of axonal transport resulting in swelling. Myelinated axons were found to be more resistant than unmyelinated ones [24].

These analyses have demonstrated that initial axotomy is not frequent in TBI, even in its severe form [24, 25]. On the other hand, axonal transection has been well documented, by electron microscopy in non-human primates, in axons of small diameter [26]. With tissue-clearing technology [25], in a controlled cortical impact model, the notion that initial axotomy is less widespread was confirmed whereby the number of swellings that appeared to be on viable axons were more numerous than expected. Their number decreased at one week and one month and there was also a decrease in their diameter. In instances of full axotomy however, their diameter appeared larger [25]. The authors described a complex array of swellings with distinct evolutions and immunophenotypes; some were negative with β-APP but positive with NF. They suggested that distinct mechanisms of degeneration were likely at play. At one month, they proposed that a number of axons were very likely salvageable, offering a window for therapeutic intervention [25]. There was no description of the neuronal perikarya in this study. A diversity of axonal abnormalities and swelling phenotypes was also reported in a non-impact rotational acceleration model of mild TBI in swine (24, 48 and 72 h post-TBI) as well as in humans (up to seven days post-TBI) by Johnson et al. [24]. Their objectives were to localize by immunohistochemistry the cellular topography of a proteolytic fragment of the axon-specific protein, spectrin, and to compare this with that of amyloid-precursor protein (APP), the gold standard for the evaluation of axonal damage, as well as of some neurofilament markers (non-phosphorylated neurofilament-H, neurofilament-68 and compacted neurofilament-medium). Beyond the variety of immunohistochemical phenotypes, no neuronal somatic changes were reported in the human brains (note however that the post-TBI period of the study did not extend beyond seven days) [24]. Assessment of the presence of proximal swellings was not included in the study.

The mechanisms leading to the ballooning of neuronal somata described in this retrospective descriptive study remain unknown. In line with experimental findings reported by Singleton et al. [18], we were able to demonstrate proximal axonal swellings in the cingulum and hippocampal structures, a traumatic complication that has not attracted significant attention in the literature. One could postulate that this is a common finding in severe TBI. The prevalence of these axonal swellings is not commensurate with the relative paucity of ballooned neurons in severe TBI patients, as reported here. It is worth noting again that the morphological detection of these ballooned neurons was done weeks to months after the impact. It is conceivable that factors related to selective neuronal vulnerability, a well-known concept in neurodegeneration, underlie this phenomenon wherein only a subset of neurons with damaged axons manifest a ballooned morphology. Alternatively, or in addition, it may be that ballooning requires full axonal transection, and that those neurons whose axons are merely swollen do not become "ballooned". Regardless, the neuronal changes described herein add to the notion that TBI triggers a dynamic and complex process of degeneration and regeneration.

In summary, we report the presence of ballooned neurons, predominant in the anterior cingulum, in three cases of semi-recent severe TBI with coma from traumatic incident to death. In line with experimental data, we postulate that proximal axonal damage may be needed to generate this complication. This is a small series of cases, and more studies are needed to better establish the frequency of this complication, the mechanistic and pathogenic relationship of the axonal damage and the ballooning of neurons and the possible link between this neuronal complication targeting preferentially the cingulum and the clinical symptomatology of CTE.

## Supplementary Information

**Additional file 1**. Histologic findings specific for each case.

## Data Availability

The paraffin blocks and all histological slides are stored in the file of the Laboratoire de sciences judiciaires et de médecine légale, Montréal, Québec and the Eastern Ontario Forensic Pathology Unit, Ottawa, Ontario.
