# Supplementary material for: Ballooned neurons in semi-recent severe traumatic brain injury
Source: Acta Neuropathol Commun. 2023 Mar 10;11:37. doi: 10.1186/s40478-023-01516-x (PMC9999665; doi:10.1186/s40478-023-01516-x)
Supplement: Supplementary file 1 — Additional file 1. Histologic findings specific for each case. [file 40478_2023_1516_MOESM1_ESM.docx]

Additional File 1: Histologic findings specific to each case

Case1: The histology revealed widespread and quite severe changes of either traumatic or hypoxic-ischemic nature, all with reactive microglial and glial changes consistent with the 16- day survival. Contusions were present in the orbitofrontal and temporal lobes, with post-lobectomy destructive changes in the right frontal lobe. Microvascular pathology was significant (thrombosis, pseudo-aneurysms, parietal tears), predominantly in the white matter of the frontal and temporal lobes and the arachnoid, some with reorganizing hemorrhage, some with iron positive siderophages only. One focus of hemorrhage was noted in the left globus pallidus. Hypoxic-ischemic changes, characterized by loss of neurons and focal infarcts, were seen in the frontal, parietal and temporal lobes, hippocampi, and cerebellum. There were changes consistent with Wallerian degeneration in the deep white matter and internal capsules.

Case 2: There was diffuse activation of the microglial population and generalized reactive gliosis, variable in intensity. Arteriolosclerosis was mild in the deep gray structures and moderate in the hemispheric white matter with pallor of the myelin reaction. Mild non-specific neuronal loss was noted in the frontal cerebral cortex and subiculi. Senile changes were consistent with age, with predominant primitive plaques, rare classic senile plaques and a few tau-positive neurons with moderate numbers of Hirano bodies in the hippocampi. A single cluster of sub-pial tau-positive astrocytes was present in the depth of a crest, in the right frontal lobe.

Case 3: In line with the gross findings, contusions were documented in both orbitofrontal lobes and a resorbing hematoma was identified in the left posterior temporal lobe. The white matter of the left frontotemporal and right parietal lobes showed resorbing perivascular hemorrhage consistent with diffuse microvascular injury. There was a diffuse mild microglial and astrocytic reaction, as well as focal neuronal loss in the cerebral cortex, inferior olivary nuclei, dentate nuclei, and cerebellar cortex. Subtle recent hypoxic ischemic changes were noted in the frontal cerebral cortex and hippocampi.
